# Impact of 10-Valent Pneumococcal Conjugate Vaccine Introduction on Pneumococcal Carriage and Antibiotic Susceptibility Patterns Among Children Aged <5 Years and Adults With Human Immunodeficiency Virus Infection: Kenya, 2009–2013

**DOI:** 10.1093/cid/ciz285

**Published:** 2020-02-14

**Authors:** Miwako Kobayashi, Godfrey Bigogo, Lindsay Kim, Ondari D. Mogeni, Laura M. Conklin, Arthur Odoyo, Herine Odiembo, Fabiana Pimenta, Dominic Ouma, Aaron M. Harris, Kennedy Odero, Jennifer L. Milucky, Alice Ouma, George Aol, Allan Audi, Clayton Onyango, Leonard Cosmas, Geofrey Jagero, Jennifer L. Farrar, Maria da Gloria Carvalho, Cynthia G. Whitney, Robert F. Breiman, Fernanda C. Lessa

**Affiliations:** 1 Respiratory Diseases Branch, Centers for Disease Control and Prevention, Atlanta, Georgia;; 2 Centre for Global Health Research, Kenya Medical Research Institute, Kisumu;; 3 US Public Health Service, Rockville, Maryland;; 4 Centre for Global Health Research, Nairobi, Kenya;; 5 Global Disease Detection Division, Centers for Disease Control and Prevention, Nairobi;; 6 University of Maryland School of Medicine, Center for International Health, Education, and Biosecurity Kenya Programs, Nairobi;; 7 Emory Global Health Institute, Atlanta, Georgia

**Keywords:** *Streptococcus pneumoniae*, 10-valent pneumococcal conjugate vaccine, pneumococcal carriage, catch-up vaccination, antibiotic nonsusceptibility

## Abstract

**Background.:**

Kenya introduced 10-valent pneumococcal conjugate vaccine (PCV10) among children <1 year in 2011 with catch-up vaccination among children 1–4 years in some areas. We assessed changes in pneumococcal carriage and antibiotic susceptibility patterns in children <5 years and adults.

**Methods.:**

During 2009–2013, we performed annual cross-sectional pneumococcal carriage surveys in 2 sites: Kibera (children <5 years) and Lwak (children <5 years, adults). Only Lwak had catch-up vaccination. Nasopharyngeal and oropharyngeal (adults only) swabs underwent culture for pneumococci; isolates were serotyped. Antibiotic susceptibility testing was performed on isolates from 2009 and 2013; penicillin nonsusceptible pneumococci (PNSP) was defined as penicillin-intermediate or -resistant. Changes in pneumococcal carriage by age (<1 year, 1–4 years, adults), site, and human immunodeficiency virus (HIV) status (adults only) were calculated using modified Poisson regression, with 2009–2010 as baseline.

**Results.:**

We enrolled 2962 children (2073 in Kibera, 889 in Lwak) and 2590 adults (2028 HIV+, 562 HIV−). In 2013, PCV10-type carriage was 10.3% (Lwak) to 14.6% (Kibera) in children <1 year and 13.8% (Lwak) to 18.7% (Kibera) in children 1–4 years. This represents reductions of 60% and 63% among children <1 year and 52% and 60% among children 1–4 years in Kibera and Lwak, respectively. In adults, PCV10-type carriage decreased from 12.9% to 2.8% (HIV+) and from 11.8% to 0.7% (HIV−). Approximately 80% of isolates were PNSP, both in 2009 and 2013.

**Conclusions.:**

PCV10-type carriage declined in children <5 years and adults post–PCV10 introduction. However, PCV10-type and PNSP carriage persisted in children regardless of catch-up vaccination.

Pneumonia remains the leading cause of deaths in children aged less than 5 years worldwide; and in 2015, *Streptococcus pneumoniae* was the most common etiology of pneumonia-related deaths [1]. The World Health Organization (WHO) recommends inclusion of pneumococcal conjugate vaccine (PCV) in childhood immunization programs [2]. With support of Gavi, the Vaccine Alliance, 33 out of 37 Gavi-supported countries in Africa introduced PCV by 2015 [3].

Kenya introduced 10-valent PCV (PCV10) into the routine national immunization program in February 2011 using a 3-primary-doses-without-a-booster (3 + 0) schedule. During the first year of introduction, all children aged less than 1 year were targeted to receive 3 doses. Catch-up vaccination campaigns took place in selected sites, where children aged 1–4 years received up to 2 doses of PCV10. After the first year of introduction, PCV10 was only offered following the routine immunization schedule, with doses given to infants at 6, 10, and 14 weeks. The impact of PCV introduction against pneumococcal carriage and invasive pneumococcal disease (IPD) has been well documented [4–7]; however, as one of the first countries in Africa to introduce PCV10, it was unclear whether the significant impact seen in other settings would be observed in Kenya, given the differences in pneumococcal carriage, serotype distribution, and factors associated with carriage (eg, household settings, prevalence of underlying conditions). Therefore, annual cross-sectional pneumococcal carriage surveys were conducted pre– and post–PCV10 introduction in sites with and without catch-up vaccination among children aged 1–4 years. The primary objective was to assess the impact of PCV10 introduction in Kenya on pneumococcal carriage among children aged less than 5 years targeted for vaccination and adults in the same household who were not targeted for vaccination. Our secondary objectives were as follows: 1) to compare the impact of different PCV10 introduction strategies on carriage, 2) to evaluate differences in pneumococcal carriage prevalence among adults living with and without human immunodeficiency virus (HIV) infection, and 3) to evaluate changes in the proportion of antibiotic-nonsusceptible pneumococci among carried strains.

## METHODS

### Study Setting

We utilized 2 ongoing surveillance platforms to conduct annual pneumococcal carriage surveys: the Population-based Infectious Disease Surveillance (PBIDS) and the Western Kenya Health and Demographic Surveillance System (HDSS). PBIDS is conducted in 2 geographically distinct regions, Kibera and Lwak [8, 9]. Catch-up vaccination was implemented in Lwak but not in Kibera. HDSS has been in place in western Kenya; and since 2005, residents of 33 of the HDSS villages have also been enrolled in Lwak PBIDS [10] (see Supplementary Methods).

### Cross-sectional Survey in Children

Annual cross-sectional pneumococcal carriage surveys were performed in Kibera and Lwak among children aged less than 5 years during 2009–2013, October–December. Children aged less than 5 years were sampled differently in the 2 sites: in Lwak (with catch-up vaccination targeting children aged 1–4 years), children aged less than 5 years were randomly selected; in Kibera (no catch-up vaccination), age-stratified (<1 year and 1–4 years) random sampling was performed (see Supplementary Figure). Survey methods are described in detail elsewhere [11]. Upon enrollment, trained surveyors collected information on household characteristics, respiratory illness, and antibiotic use using standardized forms. PCV10 vaccination history was obtained for children enrolled during 2011–2013. Surveyors were instructed to verify each reported vaccine dose with the child’s vaccination card. Vaccination history was validated through PBIDS and HDSS records whenever possible.

### Cross-sectional Survey in Adults

In Lwak, pneumococcal carriage surveys among adults living with children aged less than 5 years were conducted in 2009 and annually from 2011 to 2013 during October–December. Details of the survey methods were previously described [10]. Briefly, HDSS records were used to identify compounds where at least 1 HIV-positive parent of a child aged less than 5 years resided. HDSS records are linked to home-based counseling and testing for HIV that occurred widely in the area during 2008–2009 [12]. To maintain confidentiality of HIV testing status, all adults with children aged less than 5 years living in the same compound were invited to participate, regardless of HIV status. For each adult enrolled, trained survey staff used a standardized questionnaire to collect information [10].

### Laboratory Methods

Calcium alginate swabs were used for nasopharyngeal specimen (children and adults) oropharyngeal specimen (adults only) collection. Processed specimens were transported to the Kenya Medical Research Institute laboratory in Kisumu, Kenya, for pneumococcal isolation [13]. Pneumococcal isolates recovered from specimens collected from 2009 survey participants were serotyped by Quellung reaction. Isolates recovered from participants enrolled during 2010–2013 were serotyped by multiplex polymerase chain reaction–based testing. Antibiotic susceptibility testing was performed on pneumococcal isolates from 2009 and 2013 by broth microdilution (Trek Diagnostics). Antibiotic susceptibility was determined using the 2012 Clinical and Laboratory Standards Institute criteria for minimum inhibitory concentrations [11, 14, 15]. Intermediate and resistant isolates were considered nonsusceptible to the antibiotic tested (see Supplementary Methods).

### Data Management and Analysis

We performed descriptive analyses of participants by site, year, and age group (<1 year, 1–4 years, and adults). We calculated unadjusted prevalence ratios using classic methods for estimation of risk ratios with 2009–2010 (2009 only in adults) as the reference period. Potential confounders associated with pneumococcal carriage identified through previous studies were explored, and all models were adjusted for respiratory illness within the past 30 days, antibiotic use within 7 days, and area used for cooking [10, 16–18]. Adjusted prevalence ratios (aPRs) were calculated using Poisson regression with robust error variance [19]. Changes in carriage prevalence by serotype were compared between 2009 and 2013 for children aged less than 5 years and for adults. Changes in the proportion (among pneumococcal isolates) and carriage prevalence (among survey participants) of antibiotic-nonsusceptible pneumococci between 2009 and 2013 were tested. Chi-square test or Fisher’s exact test was used to compare categorical variables and Wilcoxon rank-sum test was used for continuous variables. Analyses were performed using SAS software (version 9.4; SAS Institute) (see Supplementary Methods).

### Ethical Considerations

The study was approved by the ethics committees at the Kenya Medical Research Institute and the Centers for Disease Control and Prevention. Written informed consent was obtained from all adult participants and the parent or guardian of all participating children.

### Definitions

See the Supplementary Methods for definitions on PCV10-type, 13-valent PCV (PCV13)-unique-type, nonvaccine-type (NVT), and antibiotic susceptibility determinations.

## RESULTS

### Overall Characteristics in Children

During 2009–2013, 2073 children in Kibera and 889 children in Lwak were enrolled (Table 1). Kibera children were more likely to sleep in a crowded room (median number of people sleeping in the same room, 5 vs 3; *P* < .001) and report recent (≤30 days of the survey) respiratory illness than Lwak children (67.3% vs 56.7%, *P* < .001). Kibera children were less likely than Lwak children to be exposed to tobacco smoke at home (10.2% vs 15.8%, *P* < .001) and have recent (≤7 days before the survey) antibiotic use (18.3% vs 22.4%, *P* = .01). In both sites, about one-third of participants reported taking antibiotics within 30 days of the survey; cotrimoxazole and penicillin/amoxicillin were the most frequently reported antibiotics. Differences in types of fuel used and area used for cooking were also noted between the sites. Characteristics of children by survey year are summarized in Supplementary Tables 1 and 2.

### Overall Characteristics in Adults

In 2009 and during 2011–2013, a total of 3547 adults were recruited in Lwak. Of those, 2590 (73.0%) had known HIV status (2028 [78.3%] HIV positive and 562 [21.7%] HIV negative) and were further analyzed (Table 2). Overall, median age was 33 years (interquartile range, 28–38 years) and 67.0% were female. HIV-positive adults were older than HIV-negative adults (median age, 34 vs 30 years; *P* < .001) and more likely to report antibiotic use within 30 days of the survey, particularly cotrimoxazole (68.7% vs 15.5%, *P* < .001). Characteristics of HIV-positive adults by survey year are summarized in Supplementary Table 3.

### Vaccine Coverage in Children

Vaccination history was available for 96.0% (1734 of 1806) of children enrolled during 2011–2013 (Supplementary Tables 1 and 2), of which 60.8% (1055 of 1734) was validated. Thus, we considered any vaccine dose given to children prior to the survey as valid regardless of when it was administered. Among children aged less than 1 year, coverage for 2 or more PCV10 doses increased from 87% (2011) to 95% (2013) in Kibera and from 77% (2011) to 100% (2013) in Lwak (Table 3). Among children aged 1–4 years, only 10% in Kibera had received 2 or more PCV10 doses in the 2011 survey compared with 59% in Lwak (Table 3). By 2013, the proportion who received 2 or more PCV10 doses increased to 61% and 82% in Kibera and Lwak, respectively.

### Changes in Overall Pneumococcal Carriage Prevalence in Children and Adults

In children, overall pneumococcal carriage prevalence at baseline (2009–2010) was 87.2% (Lwak, aged 1–4 years) to 95.0% (Lwak, aged <1 years), and no significant changes were noted after vaccine introduction (Table 3). In adults, overall pneumococcal carriage prevalence decreased significantly in HIV-positive adults (43.2% to 28.1%; aPR, 0.66; 95% confidence interval [CI], 0.56–0.78) but not in HIV-negative adults (26.8% to 21.5%; aPR, 0.82; 95% CI, 0.54–1.24; Table 3).

### Changes in PCV10-Type Pneumococcal Carriage Prevalence in Children and Adults

**Figure 1.**
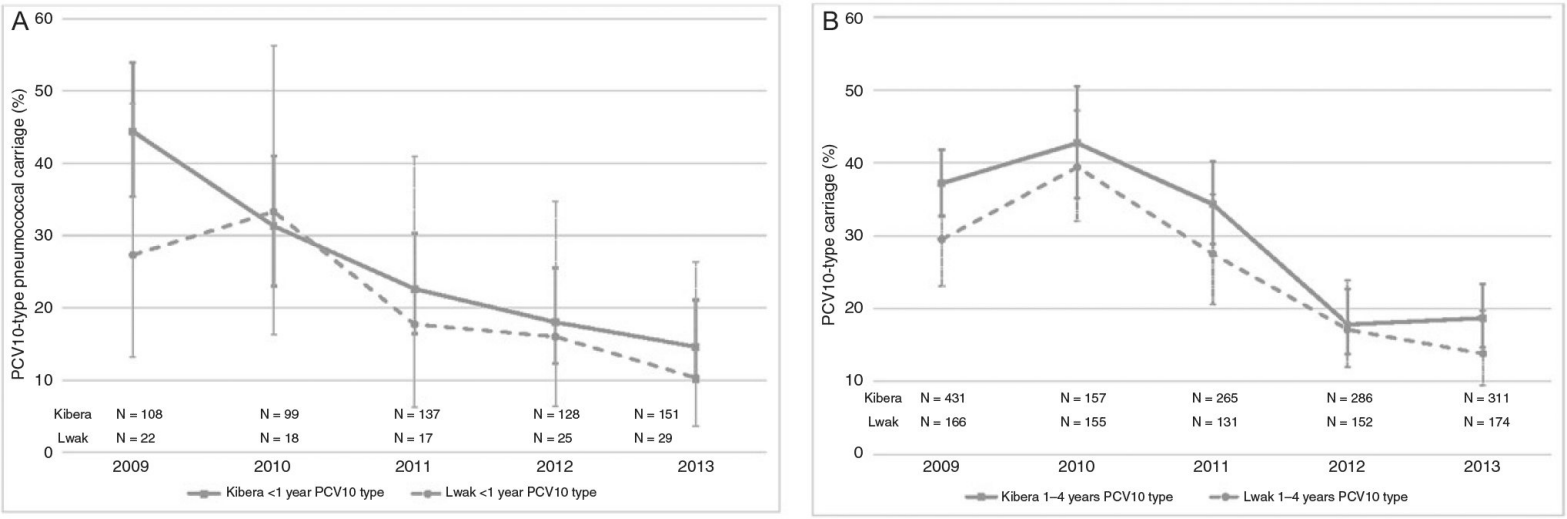
Trends in PCV10-type pneumococcal carriage prevalence, Kenya 2009–2013. Children aged <1 year in Kibera and Lwak (*A*) and children aged 1–4 years in Kibera and Lwak (*B*). Abbreviation: PCV10, 10-valent pneumococcal conjugate vaccine.

At baseline, PCV10-type carriage prevalences in children aged less than 1 year in Kibera and Lwak were 38.2% and 30.0%, respectively, and declined to 14.6% and 10.3% in 2013 (aPR in Kibera, 0.40; 95% CI, 0.26–0.62; aPR in Lwak, 0.37; 95% CI, 0.11–1.24) (Table 3, Figure 1A). For children aged 1–4 years, baseline PCV10-type carriage prevalences in Kibera and Lwak were 38.6% and 34.3%, respectively, and declined to 18.7% and 13.8% in 2013. The decline was somewhat larger in Lwak, but the CIs for changes in the 2 sites overlapped (aPR in Kibera, 0.48; 95% CI, 0.37–0.62; aPR in Lwak, 0.40; 95% CI, 0.27–0.60). There were no significant differences between sites in change in PCV10-type carriage when stratified by 1 or less PCV10 dose versus 2 or more PCV10 doses (Table 3). Unlike Kibera, carriage prevalence in children aged 1–4 years in Lwak appeared to decrease further between 2012 and 2013, but the changes were not significant (aPR, 0.84; 95% CI, 0.51–1.39) (Figure 1B). In adults, a significant reduction in 2013 compared with baseline was noted in both HIV-positive (12.9% to 2.8%; aPR, 0.24; 95% CI, 0.14–0.41) and HIV-negative (11.8% to 0.7%; aPR, 0.07; 95% CI, 0.01–0.51) adults.

### Changes in PCV13-Unique Type and NVT Pneumococcal Carriage Prevalence in Children and Adults

Compared with baseline, PCV13-unique type (serotypes contained in PCV13 but not in PCV10—3, 6A, 19A) carriage prevalence in 2013 increased in children in Kibera and HIV-positive adults, but the change was significant only in Kibera children aged 1–4 years (aPR, 1.70; 95% CI, 1.21–2.41) (Table 3). NVT carriage prevalence increased in all groups of children, although was nonsignificant in Lwak children aged less than 1 year. In contrast, NVT carriage prevalence decreased in HIV-positive adults (aPR, 0.76; 95% CI, 0.60–0.96) (Table 3).

### Changes in Serotype Distribution, 2009–2013

**Figure 2.**
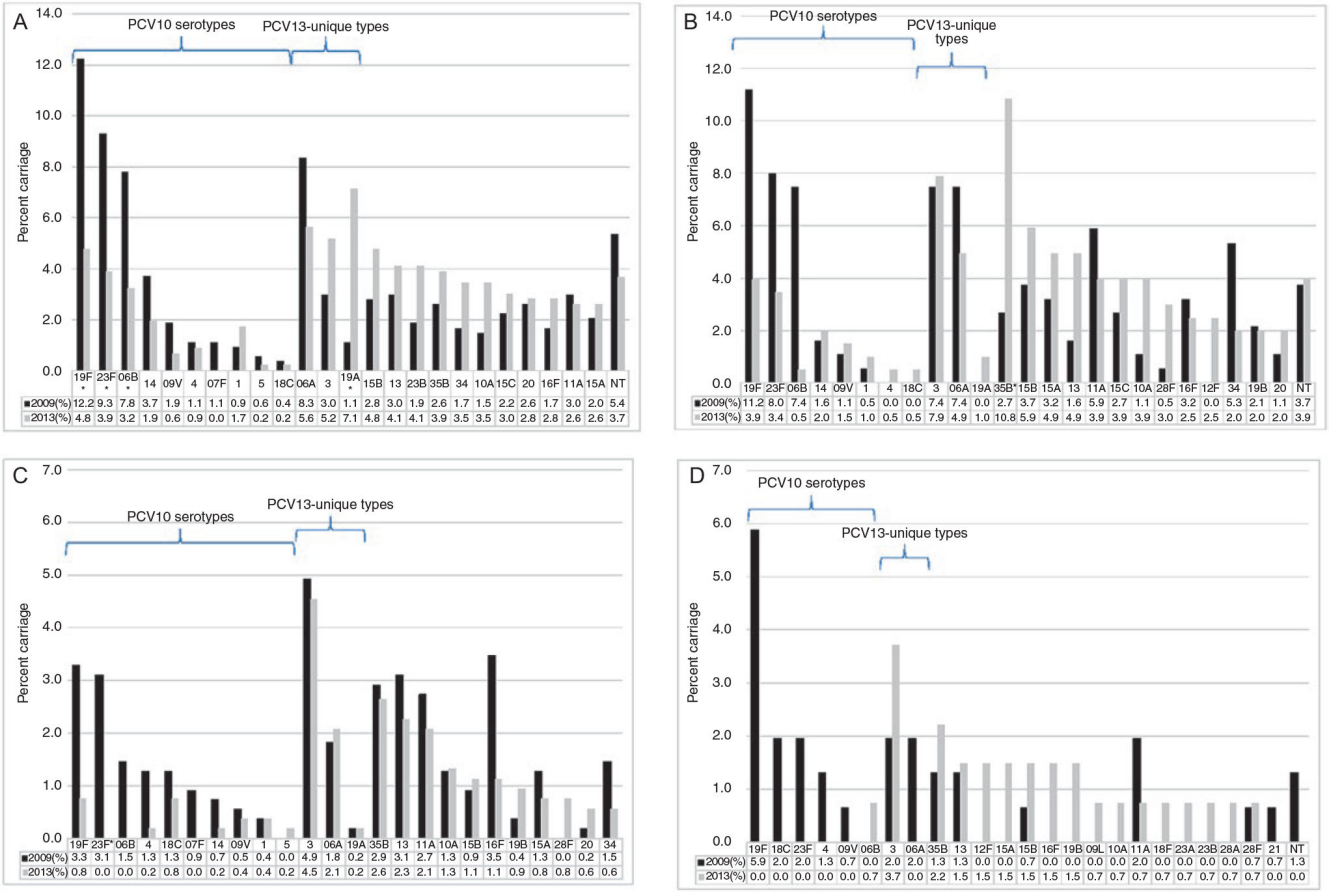
Pneumococcal carriage prevalence by serotype before (2009) and after (2013) PCV10 introduction. *A*, Children in Kibera aged <5 years, 2009 (N = 539) and 2013 (N = 462). *B*, Children in Lwak aged <5 years, 2009 (N = 188) and 2013 (N = 203). *C*, HIV-positive adults, 2009 (N = 549) and 2013 (N = 530). *D*, HIV-negative adults, 2009 (N = 153) and 2013 (N = 135). Abbreviations: HIV, human immunodeficiency virus; NT, non-typeable; PCV10, 10-valent pneumococcal conjugate vaccine; PCV13, 13-valent pneumococcal conjugate vaccine. *P < .002.

Among Kibera children aged less than 5 years, serotypes 19F, 23F, and 6B (PCV10 types) decreased significantly, while serotype 19A (PCV13-unique type) increased from 1.1% to 7.1% (*P* < .001) (Figure 2A). Among Lwak children, the decline was only significant for serotype 6B among the individual PCV10 types (Figure 2B). There was no significant change in the individual PCV13-unique types. Among NVT, serotype 35B increased significantly and was the predominant serotype in 2013.

In adults, the predominant serotypes in 2009 included PCV10 serotypes 19F and 23F along with PCV13-unique types 3 and 6A, similar to Lwak children (Figure 2C, D). In 2013, some of the PCV10 types were not detected in HIV-positive or HIV-negative adults. In HIV-positive adults, serotype 3 (PCV13-unique type) carriage remained stable and was the most commonly identified serotype in both years (4.9% vs 4.5%, *P* = .76) (Figure 2C); the increase in serotype 3 was not significant among HIV-negative adults (2.0% vs 3.7%, *P* = .37).

### Changes in the Proportion of Antibiotic Nonsusceptible Pneumococcal Isolates

Antimicrobial susceptibility testing results were available from a total of 1637 out of 1714 (95.5%) pneumococcal isolates collected in 2009 and 2013 (944 from Kibera, 344 from Lwak children, and 349 from HIV-positive adults) (Table 4). In both sites, age groups, and years, more than 95% of pneumococcal isolates were nonsusceptible to cotrimoxazole, and approximately 80% were penicillin nonsusceptible; the majority of penicillin-nonsusceptible isolates had minimum inhibitory concentrations in the intermediate range. Ceftriaxone-intermediate isolates, not detected in 2009, were observed among 7 isolates (5 were serotype 3, one each of serotypes 19F and 35B) from Kibera children in 2013. PCV10-type penicillin-intermediate *S. pneumoniae* (PISP) carriage prevalence declined significantly in children and HIV-positive adults (Table 5). The reduction was offset by a significant increase in PISP among NVT in children but not in HIV-positive adults.

## DISCUSSION

In children, we observed a 52% (Kibera, aged 1–4 years) to 60% (Kibera, aged <1 year; Lwak, aged 1–4 years) reduction in PCV10-type pneumococcal carriage prevalence approximately 2 years after PCV10 introduction, similar to the report from Kilifi, Kenya (site with catch-up vaccination targeting children aged 1–4 years) [4]. Reductions were also observed among adults, including those who were HIV positive. Despite reductions, PCV10-type carriage remained common in children in 2013 (15–19% in Kibera, 10–14% in Lwak). These figures are consistent with the results from Kilifi, Kenya [4], but higher than from countries that used a PCV10 schedule with 3 primary doses plus a booster (3 + 1) [20–22]. No significant reduction in penicillin nonsusceptible pneumococci (PNSP) carriage was observed except in HIV-positive adults. WHO currently recommends both the 3 + 0 and 2 primary doses and a booster (2 + 1) vaccination schedule for PCV administration for infants [23]. Although the 3 + 0 schedule is widely used in sub-Saharan Africa, data from carriage [24] and IPD [25] studies suggest that the booster dose in the 2 + 1 schedule might be required to achieve a sustained reduction in vaccine-type colonization. While this might explain the residual PCV10-type carriage that we observed in children, the long-term significance of this finding is unknown. In Kilifi, PCV10-type carriage in children aged less than 5 years remained at 6% 6 years after PCV10 introduction [26], yet a significant reduction in PCV10-type IPD in all age groups including unvaccinated adults was observed (92% reduction in children aged <5 years, 74% in children aged 5–14 years, and 81% in those aged ≥15 years) [27]. Whether this impact was blunted due to persistent circulation of PCV10-type pneumococci or waning mucosal immunity due to lack of a booster PCV dose is unknown. Of note, our results showed that PCV10-type carriage in 2013 was close to elimination in HIV-negative adults.

Of the PCV13-unique types, we observed a significant increase in serotype 19A carriage prevalence post–PCV10 introduction in Kibera. Although the serotype 19F antigen contained in both the 7-valent PCV (PCV7) and PCV10 was thought to provide some cross-protection against serotype 19A [28], cross-protection has not been proven and increases in serotype 19A IPD incidence post–PCV7 introduction were reported in multiple countries [29–31]. In our study, the increase in carriage of 19A was nonsignificant among children in Lwak and was not observed in adults. Carriage studies conducted in Kilifi [4] and Brazil [20] within 3 years of PCV10 introduction (both with catch-up vaccination targeting the studied age groups) also reported nonsignificant increases in serotype 19A carriage post–PCV10 introduction. Although Brazil reported an increase in IPD due to non-PCV10 serotypes (3, 6C, and 19A) 5 years after PCV10 introduction, results from Kilifi showed nonsignificant results [27]; and so far, longer-term impact of PCV10 against serotype 19A has been undetermined [32, 33]. Since our study was not powered to assess changes in individual serotypes, follow-up and correlation with IPD trends are needed.

As previously described [11], a high proportion of pneumococcal carriage isolates were nonsusceptible to penicillin and cotrimoxazole, and the proportion was essentially unchanged after PCV10 introduction. PISP carriage prevalence among PCV10 types declined significantly, but this decline was balanced by a significant increase in PISP NVT carriage in children. Similar findings were reported both in US children [7, 34] and adults [35] post–PCV7 introduction. However, isolates with intermediate susceptibility to ceftriaxone were detected for the first time in the post-PCV10 period, most of which were serotype 3. In the United States, IPD caused by NVT antibiotic-nonsusceptible *S. pneumoniae* remained below the pre-PCV period [36, 37]. In Kenya, however, the higher proportion of overall pneumococcal carriage and antibiotic use in the community might result in more sustained transmission of antibiotic-nonsusceptible *S. pneumoniae*. Although antibiotic susceptibility results were not available, data from Kilifi showed a nonsignificant increase in NVT-IPD in all age groups aged 2 or more months, despite significant reductions in overall and PCV10-type IPD incidence [27].

We observed a reduction in overall pneumococcal carriage prevalence and PNSP carriage prevalence among HIV-positive adults post–PCV10 introduction. This is likely due to the significant reduction in NVT carriage prevalence observed in 2013, but not in 2011 or 2012, compared with baseline. Similar findings have been reported [38] and attributed to different dynamics in pneumococcal carriage (HIV-positive mothers are more likely to carry vaccine-type pneumococci) [39], changes in HIV management over time, and widespread use of antibiotic prophylaxis [40, 41]. Despite the observed reductions, the high proportion of serotype 3 carriage, a serotype known to be associated with severe outcomes [42], and the higher carriage prevalence compared with HIV-negative adults indicate that HIV-positive adults will continue to be at a higher risk of IPD than HIV-negative adults, as seen in other countries [40, 43].

Our study is subject to several limitations. First, we were not able to assess differences in indirect effects between Lwak and Kibera due to the small number of unvaccinated Lwak children aged 1–4 years. We used children who received 1 or less PCV10 dose as a proxy for unvaccinated children, but the number of children in this group was also small. Since adults were only recruited in Lwak, we could not compare indirect effects among adults between the 2 sites. Second, although we were not able to confirm PCV10 vaccination dates for all children, we considered any vaccine dose reported prior to the survey regardless of the timing; therefore, some reported doses might not have been valid. Third, misclassification of HIV-negative adults might have occurred since some adults had a test date more than 1 year before the survey. Last, we did not have data on CD4 counts or HIV treatment history when assessing the impact of PCV10 among HIV-positive adults.

Despite these limitations, our study addresses key questions related to PCV10 introduction in sub-Saharan Africa. We observed significant declines in PCV10-type carriage in children and adults approximately 2 years after PCV10 introduction in Kenya. Although not statistically significant, there was a greater reduction in PCV10-type carriage in the site with catch-up vaccination targeting older children compared with the site without catch-up vaccination. However, as additional birth cohorts receive vaccination and as vaccinated children age, the differences between the 2 sites might be less noticeable. We did not observe changes in the proportion of isolates that were PNSP in children post-PCV10; this is likely due to rapid replacement of PCV10-type isolates by NVT PNSP. Very few studies have comprehensively addressed the impact of PCV10 introduction in sub-Saharan Africa, where 3 + 0 is widely used. The most recent WHO position statement on PCV states the potential benefits of the 2 + 1 schedule over the 3 + 0 schedule in providing longer protection, although supporting data are currently limited [23]. Continued monitoring of PCV10-type pneumococcal carriage in children and antibiotic-nonsusceptible pneumococci and their association with disease rates could help inform pneumococcal vaccination policies.

## Supplementary Material

Supplemental file

## Figures and Tables

**Table 1.** Characteristics of Survey Participants Aged <5 Years in Kibera and Lwak, Kenya: 2009–2013

	Kibera (N = 2073)	Lwak (N = 889)	*P* Value
Age, n (%)			
<1 year	623 (30.1)	111 (12.5)	<.001
1–4 years	1450 (69.9)	778 (87.5)	
Females, n (%)	1053 (50.8)	438 (49.3)	.446
Number of people sleeping in the same room as the child, median (IQR)	5 (4–6)	3 (3–4)	<.001
Number of children in the household attending school or daycare, n (%)			
0	370 (17.9)	118 (13.3)	.002
≥1	1700 (82.1)	771 (86.7)	
Cough, fever, fast breathing, or pneumonia within 30 days of the survey, n (%)	1394 (67.3)	504 (56.7)	<.001
Tobacco smoke at home, n (%)	212 (10.2)	140 (15.8)	<.001
Types of fuels used for cooking, n (%)			
Fuelwood	22 (1.1)	862 (97.0)	<.001
Charcoal	1968 (94.9)	353 (39.7)	<.001
Kerosene	1421 (68.6)	32 (3.6)	<.001
Others	67 (3.2)	22 (2.5)	.269
Area used for cooking, n (%)			
Same area where you live or sleep	1923 (92.8)	244 (27.5)	<.001
All other sites	150 (7.2)	645 (72.6)	
Any antibiotic use, n (%)			
Current use	132 (6.4)	77 (8.7)	.026
Use within 7 days of the survey	379 (18.3)	199 (22.4)	.010
Use within 30 days of the survey	780 (37.6)	332 (37.4)	.885
Use of cotrimoxazole, n (%)			
Current use^a^	62 (3.0)	54 (6.1)	<.001
Use within 7 days of the survey^b^	162 (7.8)	148 (16.7)	<.001
Use within 30 days of the survey^c^	290 (14.1)	190 (21.6)	<.001
Use of penicillin or amoxicillin, n (%)			
Current use^d^	65 (3.1)	14 (1.6)	.017
Use within 7 days of the survey^e^	219 (10.6)	49 (5.5)	<.001
Use within 30 days of the survey^f^	502 (24.3)	102 (11.6)	<.001

Abbreviation: IQR, interquartile range.

a Excluded 8 who responded “don’t know.”

b Excluded 8 who responded “don’t know.”

c Excluded 21 who responded “don’t know.”

d Excluded 9 who responded “don’t know.”

e Excluded 7 who responded “don’t know.”

f Excluded 16 who responded “don’t know.”

**Table 2.** Characteristics of Adult Participants in Lwak by Human Immunodeficiency Virus Status: 2009, 2011–2013

	Total (N = 2590)	HIV Positive (n = 2028)	HIV Negative (n = 562)	*P* Value (HIV Positive vs HIV Negative)
Females, n (%)	1734 (67.0)	1349 (66.5)	385 (68.5)	.375
Median age (IQR), y	33 (28–38)	34 (29–39)	30 (25–37)	<.001
Aged <30 years, n (%)	838 (32.4)	581 (28.7)	257 (45.7)	<.001
Aged 30–39 years, n (%)	1224 (47.3)	1013 (50.0)	211 (37.5)	
Aged ≥40 years, n (%)	527 (30.4)	433 (21.4)	94 (16.7)	
Employed, n (%)	429 (16.0)	342 (16.9)	87 (15.5)	.425
Number of children aged <5 years in the household, median (range)	1 (1–6)	1 (1–6)	1 (1–4)	<.001
Cough, fever, fast breathing, or pneumonia within 30 days, n (%)	1233 (47.6)	984 (48.5)	249 (44.3)	.077
Tobacco smoker,^a^ n (%)	127 (4.9)	92 (4.6)	35 (6.2)	.098
Tobacco smoke at home,^b^ n (%)	341 (13.2)	244 (12.1)	97 (17.3)	.001
Any antibiotic use, n (%)				
Current use	998 (38.5)	966 (47.6)	32 (5.7)	<.001
Use within 7 days of the survey	1499 (57.9)	1405 (69.3)	94 (16.7)	<.001
Use within 30 days of the survey	1658 (64.0)	1508 (74.4)	150 (26.7)	<.001
Use of cotrimoxazole, n (%)				
Current use^c^	960 (37.1)	936 (46.2)	24 (4.3)	<.001
Use within 7 days of the survey^d^	1404 (54.2)	1349 (66.5)	55 (9.8)	<.001
Use within 30 days of the survey^e^	1481 (57.2)	1394 (68.7)	87 (15.5)	<.001
Use of penicillin or amoxicillin, n (%)				
Current use^f^	31 (1.2)	28 (1.4)	3 (0.5)	.103
Use within 7 days of the survey^g^	130 (5.0)	109 (5.4)	21 (3.7)	.114
Use within 30 days of the survey^h^	240 (9.3)	201 (9.9)	39 (6.9)	.030

Abbreviations: HIV, human immunodeficiency virus; IQR, interquartile range.

a Excluded 13 who responded “don’t know” or had a missing response.

b Excluded 8 who responded “don’t know” or had a missing response.

c Excluded 5 who responded “don’t know.”

d Excluded 1 who responded “don’t know.”

e Excluded 6 who responded “don’t know.”

f Excluded 17 who responded “don’t know.”

g Excluded 7 who responded “don’t’ know.”

h Excluded 15 who responded “don’t know.”

**Table 3.** Changes in Pneumococcal Carriage by Year, Participant Group, and 10-valent Pneumococcal Conjugate Vaccine Dose: Kibera and Lwak, 2009–2013

		Pneumococcal Carriage			
Site, Participant Group, and Year	Coverage of ≥2 PCV10 Doses, %	n/N	%	Crude PR (95% CI)	Adjusted PR (95% CI)^a^
Pneumococcal carriage, all participants^b^					
Kibera, aged <1 year					
2009–2010	…	193/207	93.2	Ref	Ref
2011	87.3	119/137	86.9	0.93 (.86, 1.00)	0.93 (.87, 1.01)
2012	91.1	121/128	94.5	1.01 (.96, 1.07)	1.01 (.96, 1.07)
2013	94.6	135/151	89.4	0.96 (.90, 1.02)	0.97 (.91, 1.04)
Lwak, aged <1 year					
2009–2010	…	38/40	95.0	Ref	Ref
2011	76.5	15/17	88.2	0.93 (.77, 1.12)	0.93 (.77, 1.14)
2012	70.8	20/25	80.0	0.84 (.68, 1.04)	0.84 (.68, 1.04)
2013	100	26/29	89.7	0.94 (.82, 1.09)	0.94 (.82, 1.09)
Kibera, aged 1–4 years					
2009–2010	…	535/588	91.0	Ref	Ref
2011	10.0	244/265	92.1	1.01 (.97, 1.06)	1.01 (.97, 1.06)
2012	35.0	266/286	93.0	1.02 (.98, 1.06)	1.02 (.98, 1.06)
2013	60.7	294/311	94.5	1.04 (1.00, 1.08)	1.04 (1.00, 1.08)
Lwak, aged 1–4 years					
2009–2010	…	280/321	87.2	Ref	Ref
2011	59.2	105/131	80.2	0.92 (.84, 1.01)	0.92 (.84, 1.02)
2012	66.7	138/152	90.8	1.04 (.97, 1.11)	1.04 (.98, 1.11)
2013	82.3	148/174	85.1	0.98 (.90, 1.05)	0.98 (.91, 1.05)
HIV-positive adults					
2009	…	237/549	43.2	Ref	Ref
2011	…	167/423	39.5	0.91 (.79, 1.06)	0.95 (.81, 1.11)
2012	…	246/526	46.8	1.08 (.95, 1.24)	1.10 (.96, 1.26)
2013	…	149/530	28.1	0.65 (.55, .77)	0.66 (.56, .78)
HIV-negative adults					
2009	…	41/153	26.8	Ref	Ref
2011	…	58/143	40.6	1.51 (1.09, 2.10)	1.52 (1.09, 2.12)
2012	…	45/131	34.4	1.28 (.90, 1.82)	1.31 (.92, 1.87)
2013	…	29/135	21.5	0.80 (.53, 1.21)	0.82 (.54, 1.24)
PCV10-type carriage, all participants^b^					
Kibera, aged <1 year					
2009–2010	…	79/207	38.2	Ref	Ref
2011	87.3	31/137	22.6	0.59 (.42, .85)	0.59 (.41, .85)
2012	91.1	23/128	18.0	0.47 (.31, .71)	0.47 (.31, .71)
2013	94.6	22/151	14.6	0.38 (.25, .58)	0.40 (.26, .62)
Lwak, aged <1 year					
2009–2010	…	12/40	30.0	Ref	Ref
2011	76.5	3/17	17.7	0.59 (.19, 1.82)	0.73 (.26, 2.02)
2012	70.8	4/25	16.0	0.53 (.19, 1.47)	0.58 (0.22, 1.50)
2013	100	3/29	10.3	0.34 (.11, 1.11)	0.37 (.11, 1.24)
Kibera, aged 1–4 years					
2009–2010	…	227/588	38.6	Ref	Ref
2011	10.0	91/265	34.3	0.89 (.73, 1.08)	0.88 (.73, 1.08)
2012	35.0	51/286	17.8	0.46 (.35, .60)	0.47 (.36, .61)
2013	60.7	58/311	18.7	0.48 (.37, .62)	0.48 (.37, .62)
Lwak, aged 1–4 years					
2009–2010	…	110/321	34.3	Ref	Ref
2011	59.2	36/131	27.5	0.80 (.58, 1.10)	0.81 (.59, 1.12)
2012	66.7	26/152	17.1	0.50 (.34, .73)	0.50 (.34, .74)
2013	82.3	24/174	13.8	0.40 (.27, .60)	0.40 (.27, .60)
HIV-positive adults					
2009	…	71/549	12.9	Ref	Ref
2011	…	33/423	7.8	0.60 (.41, .89)	0.65 (.44, .96)
2012	…	34/526	6.5	0.50 (.34, .74)	0.54 (.36, .80)
2013	…	15/530	2.8	0.22 (.13, .38)	0.24 (.14, .41)
HIV-negative adults					
2009	…	18/153	11.8	Ref	Ref
2011	…	18/143	12.6	1.07 (.58, 1.97)	1.11 (.60, 2.06)
2012	…	9/131	6.9	0.58 (.27, 1.26)	0.63 (.30, 1.34)
2013	…	1/135	0.7	0.06 (.01, .47)	0.07 (.01, .51)
PCV10-type carriage, ≤1 PCV10 dose^b^					
Kibera, aged 1–4 years					
2009–2010	…	227/588	38.6	Ref	Ref
2011	…	83/239	34.7	0.90 (.74, 1.10)	0.90 (.73, 1.10)
2012	…	37/190	19.5	0.50 (.37, .69)	0.51 (.38, .70)
2013	…	29/138	21.0	0.54 (.39, .76)	0.54 (.38, .76)
Lwak, aged 1–4 years					
2009–2010	…	110/321	34.3	Ref	Ref
2011	…	19/54	35.2	1.03 (.69, 1.52)	1.01 (.68, 1.52)
2012	…	15/54	27.8	0.81 (.51, 1.28)	0.81 (.51, 1.28)
2013	…	6/39	15.4	0.45 (.21, .95)	0.45 (.21, .96)
PCV10-type carriage, ≥2 PCV10 doses^b^					
Kibera, aged <1 year					
2009–2010	…	79/207	38.2	Ref	Ref
2011	…	29/117	24.8	0.65 (.45, .93)	0.66 (.46, .95)
2012	…	22/113	19.5	0.51 (.34, .77)	0.51 (.35, .77)
2013	…	22/139	15.8	0.41 (.27, .63)	0.44 (.29, .67)
Lwak, aged <1 year					
2009–2010	…	12/40	30.0	Ref	Ref
2011	…	2/13	15.3	0.51 (.13, 2.00)	0.66 (.18, 2.40)
2012	…	3/17	17.7	0.59 (.19, 1.82)	0.75 (.25, 2.19)
2013	…	3/29	10.3	0.34 (.11, 1.11)	0.38 (.12, 1.25)
Kibera, aged 1–4 years					
2009–2010	…	227/588	38.6	Ref	Ref
2011	…	8/26	30.8	0.80 (.44, 1.43)	0.81 (.45, 1.46)
2012	…	14/96	14.6	0.38 (.23, .62)	0.39 (.23, .63)
2013	…	29/173	16.8	0.43 (.31, .61)	0.43 (.30, .60)
Lwak, aged 1–4 years					
2009–2010	…	110/321	34.3	Ref	Ref
2011	…	17/77	22.1	0.64 (.41, 1.01)	0.66 (.42, 1.03)
2012	…	11/98	11.2	0.33 (.18, .58)	0.33 (.18, .59)
2013	…	18/135	13.3	0.39 (.25, .61)	0.38 (.24, .59)
PCV13-unique type (serotypes 3, 6A, 19A) carriage, all participants^b^					
Kibera, aged <1 year					
2009–2010	…	26/207	12.6	Ref	Ref
2011	87.3	15/137	11.0	0.87 (.48, 1.58)	0.86 (.48, 1.55)
2012	91.1	18/128	14.1	1.12 (.64, 1.96)	1.11 (.64, 1.93)
2013	94.6	28/151	18.5	1.48 (.90, 2.41)	1.38 (.84, 2.28)
Lwak, aged <1 year					
2009–2010	…	7/40	17.5	Ref	Ref
2011	76.5	1/17	5.9	0.34 (.04, 2.53)	0.24 (.03, 2.25)
2012	70.8	3/25	12.0	0.69 (.20, 2.41)	0.57 (.17, 1.89)
2013	100	3/29	10.3	0.59 (.17, 2.09)	0.52 (.14, 1.86)
Kibera, aged 1–4 years					
2009–2010	…	59/588	10.0	Ref	Ref
2011	10.0	32/265	12.1	1.20 (.80, 1.80)	1.21 (.81, 1.82)
2012	35.0	53/286	18.5	1.85 (1.31, 2.60)	1.83 (1.30, 2.57)
2013	60.7	52/311	16.7	1.67 (1.18, 2.36)	1.70 (1.21, 2.41)
Lwak, aged 1–4 years					
2009–2010	…	43/321	13.4	Ref	Ref
2011	59.2	15/131	11.5	0.85 (.49, 1.48)	0.93 (.53, 1.63)
2012	66.7	15/152	9.9	0.74 (.42, 1.28)	0.76 (.44, 1.32)
2013	82.3	24/174	13.8	1.03 (.65, 1.64)	1.03 (.65, 1.65)
HIV-positive adults					
2009	…	37/549	6.7	Ref	Ref
2011	…	28/423	6.6	0.98 (.61, 1.58)	1.05 (.65, 1.70)
2012	…	52/526	9.9	1.47 (.98, 2.20)	1.61 (1.06, 2.44)
2013	…	36/530	6.8	1.01 (.65, 1.57)	1.13 (.71, 1.79)
HIV-negative adults					
2009	…	6/153	3.9	Ref	Ref
2011	…	5/143	3.5	0.89 (.28, 2.86)	1.07 (.34, 3.38)
2012	…	9/131	6.9	1.75 (.64, 4.79)	1.89 (.67, 5.29)
2013	…	5/135	3.7	0.94 (.29, 3.02)	0.96 (.29, 3.21)
NVT carriage, all participants^b^					
Kibera, aged <1 year					
2009–2010	…	88/207	42.5	Ref	Ref
2011	87.3	73/137	53.3	1.25 (1.00, 1.57)	1.26 (1.01, 1.58)
2012	91.1	80/128	62.5	1.47 (1.19, 1.81)	1.47 (1.19, 1.80)
2013	94.6	85/151	56.3	1.32 (1.07, 1.64)	1.35 (1.09, 1.67)
Lwak, aged <1 year					
2009–2010	…	19/40	47.5	Ref	Ref
2011	76.5	11/17	64.7	1.36 (.84, 2.20)	1.36 (.87, 2.15)
2012	70.8	13/25	52.0	1.09 (.67, 1.80)	1.10 (.68, 1.77)
2013	100	20/29	69.0	1.45 (.97, 2.18)	1.47 (.98, 2.19)
Kibera, aged 1–4 years					
2009–2010	…	249/588	42.4	Ref	Ref
2011	10.0	121/265	45.7	1.08 (.92, 1.27)	1.08 (.92, 1.27)
2012	35.0	162/286	56.6	1.34 (1.16, 1.54)	1.33 (1.16, 1.53)
2013	60.7	184/311	59.2	1.40 (1.22, 1.59)	1.40 (1.23, 1.60)
Lwak, aged 1–4 years					
2009–2010	…	127/321	39.6	Ref	Ref
2011	59.2	54/131	41.2	1.04 (.82, 1.33)	1.03 (.80, 1.32)
2012	66.7	97/152	63.8	1.61 (1.35, 1.93)	1.60 (1.34, 1.92)
2013	82.3	100/174	57.5	1.45 (1.21, 1.75)	1.46 (1.21, 1.76)
HIV-positive adults					
2009	…	129/549	23.5	Ref	Ref
2011	…	106/423	25.1	1.07 (.85, 1.33)	1.08 (.86, 1.36)
2012	…	160/526	30.4	1.29 (1.06, 1.58)	1.26 (1.03, 1.53)
2013	…	98/530	18.5	0.79 (.62, .99)	0.76 (.60, .96)
HIV-negative adults					
2009	…	17/153	11.1	Ref	Ref
2011	…	35/143	24.5	2.20 (1.29, 3.75)	2.10 (1.23, 3.58)
2012	…	27/131	20.6	1.86 (1.06, 3.25)	1.84 (1.05, 3.22)
2013	…	23/135	17.0	1.53 (.86, 2.75)	1.53 (.85, 2.72)

Abbreviations: CI, confidence interval; HIV, human immunodeficiency virus; NVT, nonvaccine type; PCV10, 10-valent pneumococcal conjugate vaccine; PCV13, 13-valent pneumococcal conjugate vaccine; PR, prevalence ratio; Ref, reference.

a Adjusted for respiratory illness within the past 30 days, antibiotic use within 7 days, and area used for cooking.

b None of the children in 2009–2010 and none of the adults received pneumococcal conjugate vaccine.

**Table 4.** Changes in Antibiotic Susceptibility Among Pneumococcal Strains Identified From Carriers Before (2009) and After (2013) 10-Valent Pneumococcal Conjugate Vaccine Introduction

	Susceptible		Intermediate		Resistant		
Antibiotic and Year	Break Point (μg/mL)	n (%)	Break Point (μg/mL)	n (%)	Break Point (μg/mL)	n (%)	*P* Value (2009 vs 2013)
Pneumococcal isolates from Kibera children aged <5 years (N = 499 in 2009, N = 445 in 2013)							
Penicillin							
2009	≤0.06	100 (20.0)	0.12–1	387 (77.6)	≥2	12 (2.4)	.618
2013	…	100 (22.5)	…	333 (74.8)	…	12 (2.7)	…
Chloramphenicol							
2009	≤4	490 (98.2)	NA	…	≥8	9 (1.8)	.182
2013	…	431 (96.9)	…	…	…	14 (3.2)	…
Levofloxacin							
2009	≤2	499 (100)	4	0	≥8	0	NA
2013	…	445 (100)	…	0	…	0	…
Erythromycin							
2009	≤0.25	490 (98.2)	0.5	1 (0.2)	≥1	8 (1.6)	.042
2013	…	428 (96.2)	…	0	…	17 (3.8)	…
Ceftriaxone							
2009	≤1	499 (100)	2	0	≥4	0	.005
2013	…	438 (98.4)	…	7 (1.6)	…	0	…
Tetracycline							
2009	≤2	401 (80.4)	4	13 (2.6)	≥8	85 (17.0)	.187
2013	…	377 (84.7)	…	11 (2.5)	…	57 (12.8)	…
Cotrimoxazole							
2009	≤0.5/9.5	15 (3.0)	1/19–2/38	54 (10.8)	≥4/76	430 (86.2)	.029
2013	…	17 (3.8)	…	27 (6.1)	…	401 (90.1)	…
Clindamycin							
2009	≤0.25	498 (99.8)	0.5	0	≥1	1 (0.2)	.001
2013	…	433 (97.3)	…	0	…	12 (2.7)	…
Pneumococcal isolates from Lwak children aged <5 years (N = 163 in 2009, N = 181 in 2013)							
Penicillin							
2009	≤0.06	27 (16.6)	0.12–1	133 (81.6)	≥2	3 (1.8)	.235
2013	…	31 (17.1)	…	150 (82.3)	…	0	…
Chloramphenicol^a^							
2009	≤4	153 (97.5)	N/A	…	≥8	4 (2.6)	.757
2013	…	175 (96.7)	…	…	…	6 (3.3)	…
Levofloxacin^a^							
2009	≤2	157 (100)	4	0	≥8	0	NA
2013	…	181 (100)	…	0	…	0	…
Erythromycin							
2009	≤0.25	162 (99.4)	0.5	0	≥1	1 (0.6)	.474
2013	…	181 (100)	…	0	…	0	…
Ceftriaxone							
2009	≤1	163 (100)	2	0	≥4	0	NA
2013	…	181 (100)	…	0	…	0	…
Tetracycline							
2009	≤2	131 (80.4)	4	2 (1.2)	≥8	30 (18.4)	.001
2013	…	143 (79.0)	…	17 (9.4)	…	21 (11.6)	…
Cotrimoxazole^b^							
2009	≤0.5/9.5	0	1/19–2/38	9 (5.6)	≥4/76	152 (94.4)	1.000
2013	…	0	…	10 (5.5)	…	171 (94.5)	…
Clindamycin							
2009	≤0.25	163 (100)	0.5	0	≥1	0	NA
2013	…	181 (100)	…	0	…	0	…
Pneumococcal isolates from HIV-positive adults (N = 199 in 2009, N = 150 in 2013)							
Penicillin							
2009	≤0.06	33 (16.6)	0.12–1	162 (81.4)	≥2	4 (2.0)	.549
2013	…	32 (21.3)	…	116 (77.3)	…	2 (1.3)	…
Chloramphenicol							
2009	≤4	196 (98.5)	N/A	…	≥8	3 (1.5)	.181
2013	…	144 (96.0)	…	…	…	6 (4.0)	…
Levofloxacin							
2009	≤2	199 (100)	4	0	≥8	0	NA
2013	…	150 (100)	…	0	…	0	…
Erythromycin							
2009	≤0.25	197 (99.0)	0.5	0	≥1	2 (1.0)	1.000
2013	…	148 (98.7)	…	0	…	2 (1.3)	…
Ceftriaxone							
2009	≤1	199 (100)	2	0	≥4	0	NA
2013	…	150 (100)	…	0	…	0	…
Tetracycline^c^							
2009	≤2	150 (75.8)	4	8 (4.0)	≥8	40 (20.2)	.534
2013	…	109 (72.7)	…	10 (6.7)	…	31 (20.7)	…
Cotrimoxazole^c^							
2009	≤0.5/9.5	1 (0.5)	1/19–2/38	6 (3.1)	≥4/76	190 (96.5)	.474
2013	…	0	…	2 (1.3)	…	148 (98.7)	…
Clindamycin							
2009	≤0.25	198 (99.5)	0.5	0	≥1	1 (0.5)	.579
2013	…	148 (98.7)	…	0	…	2 (1.3)	…

Abbreviations: HIV, human immunodeficiency virus; NA, not applicable.

a Six isolates in 2009 were missing susceptibility testing results for both chloramphenicol and levofloxacin.

b Two isolates in 2009 were missing susceptibility testing results for cotrimoxazole only.

c One isolate from 2009 was missing susceptibility testing results for both tetracycline and cotrimoxazole; 1 isolate from 2009 was missing susceptibility testing results for cotromixazole only.

**Table 5.** Changes in Carriage Prevalence of Penicillin-nonsusceptible Pneumococci in 2009 Versus 2013 by Serotype Groups

	Children in Kibera Aged <5 Years			Children in Lwak Aged <5 Years			HIV-positive Adults		
Serotype	2009 (N = 521^a^), n (%)	2013 (N = 459^b^), n (%)	*P* Value	2009 (N = 184^c^), n (%)	2013 (N = 201^d^), n (%)	*P* Value	2009 (N = 506^e^), n (%)	2013 (N = 529^f^), n (%)	*P* Value
Intermediate susceptibility to penicillin									
All PISP	366 (70.2)	312 (68.0)	.441	125 (67.9)	141 (70.1)	.639	156 (30.8)	114 (21.6)	<.001
PCV10 type	174 (33.4)	72 (15.7)	<.001	46 (25.0)	22 (10.9)	<.001	50 (9.9)	8 (1.5)	<.001
PCV13-unique	57 (10.9)	55 (12.0)	.609	23 (12.5)	25 (12.4)	.985	28 (5.5)	28 (5.3)	.864
NVT	141 (27.1)	185 (40.3)	<.001	64 (34.8)	102 (50.7)	.002	79 (15.6)	78 (14.7)	.697
Resistant to penicillin									
All PRSP	11 (2.1)	12 (2.6)	.604	3 (1.6)	0	.108	4 (0.8)	2 (0.4)	.442
PCV10 type	8 (1.5)	2 (0.4)	.115	1 (0.5)	0	.478	1 (0.2)	0	.489
PCV13-unique	1 (0.2)	11 (2.4)	.002	1 (0.5)	0	.478	1 (0.2)	2 (0.4)	1.000
NVT	2 (0.4)	0	.502	1 (0.5)	0	.478	2 (0.4)	0	.239
Total	377 (72.4)	324 (70.6)	.540	128 (69.6)	141 (70.1)	.901	160 (31.6)	116 (21.9)	<.001

Abbreviations: HIV, human immunodeficiency virus; NVT, nonvaccine type; PCV10, 10-valent pneumococcal conjugate vaccine; PCV13-unique, 3 serotypes contained in 13-valent pneumococcal conjugate vaccine but not in PCV10; PISP, penicillin-intermediate *Streptococcus pneumoniae*; PRSP, penicillin-resistant *Streptococcus pneumoniae*.

a Excludes 18 children with pneumococcal carriage who are missing penicillin susceptibility test results from all 539 enrolled children.

b Excludes 3 children with pneumococcal carriage who are missing penicillin susceptibility test results from all 462 enrolled children.

c Excludes 4 children with pneumococcal carriage who are missing penicillin susceptibility test results from all 188 enrolled children.

d Excludes 2 children with pneumococcal carriage who are missing penicillin susceptibility test results from all 203 enrolled children.

e Excludes 43 adults with pneumococcal carriage who are missing penicillin susceptibility test results from all 549 enrolled adults.

f Excludes 1 adult with pneumococcal carriage who are missing penicillin susceptibility test results from all 530 enrolled adults.
