# Supplementary material for: Impact of 10-Valent Pneumococcal Conjugate Vaccine Introduction on Pneumococcal Carriage and Antibiotic Susceptibility Patterns Among Children Aged <5 Years and Adults With Human Immunodeficiency Virus Infection: Kenya, 2009–2013
Source: Clin Infect Dis. Author manuscript; Available in PMC 2020 May 4. (PMC6942635; doi:10.1093/cid/ciz285)
Supplement: Supplemental file [file NIHMS1062834-supplement-Supplemental_file.docx]

**Impact of 10-valent Pneumococcal Conjugate Vaccine Introduction on Pneumococcal Carriage and Antibiotic Susceptibility Patterns among Children aged <5 Years and Adults with HIV Infection, Kenya 2009–2013**

**ONLINE SUPPLEMENT**

**Supplementary Methods: Additional Details**

*Study setting*

*Laboratory methods*

*Definitions*

*Data management and analysis*

**Supplementary Tables and Figures**

Supplementary Table 1. Characteristics of survey participants aged <5 years in Kibera by year, Kenya 2009–2013

Supplementary Table 2. Characteristics of survey participants aged <5 years in Lwak by year, Kenya 2009–2013

Supplementary Table 3. Characteristics of HIV-positive adult survey participants in Lwak by year, Kenya 2009, 2011–2013

Supplementary Figure.

**Supplementary Methods: Additional Details**

*Study setting*

We utilized two ongoing surveillance platforms to conduct annual pneumococcal carriage surveys: Population-Based Infectious Disease Surveillance (PBIDS) and western Kenya Health and Demographic Surveillance System (HDSS). PBIDS is conducted in two geographically distinct regions, Kibera and Lwak [1, 2]. Kibera is a densely-populated urban settlement in Nairobi, Kenya. Lwak is located in rural western Kenya; its population is dispersed and consists predominantly of subsistence farmers and fishermen [2]. In 2008, HIV prevalence among adults aged ≥18 years was 14.8% for Kibera and 17.4% in Lwak [3]. HDSS has been in place in western Kenya, and since 2005, residents of 33 of the HDSS villages have also been enrolled in Lwak PBIDS [4]. Methods and characteristics of both surveillance systems are described in detail elsewhere [1, 2, 5].

*Laboratory methods*

Trained study nurses collected nasopharyngeal (NP) specimens from the posterior nasopharynx in children and adults. In adults, oropharyngeal (OP) specimens were also collected to increase pneumococcus recovery [4]. Calcium alginate swabs were used for collection and each swab was immediately placed in 1.0ml skim milk-tryptone-glucose-glycerol transport medium and processed as previously described [4, 6]. Specimens were transported to the Kenya Medical Research Institute (KEMRI) laboratory in Kisumu, Kenya, for pneumococcal isolation using previously described methods [7]. If more than one potential pneumococcal colony type was identified per plate, representative samples of each colony morphology were selected for further testing. Pneumococcal isolates recovered from specimens collected from 2009 survey participants were sent to CDC’s *Streptococcus* laboratory in Atlanta, Georgia, for serotyping by Quellung reaction. For isolates recovered from participants enrolled during 2010–2013, multiplex polymerase-chain-reaction (PCR)-based serotype testing was conducted at KEMRI. All specimens that were culture-negative and all pneumococcal isolates that were PCV10-type, ungroupable (e.g., 6A/6B, 9A/9V), or negative by multiplex PCR serotyping deduction were sent to CDC’s *Streptococcus* laboratory for confirmation by culture or Quellung reaction. Antibiotic susceptibility testing was performed on pneumococcal isolates from 2009 and 2013. Antibiotic susceptibility testing was performed at KEMRI or CDC by broth microdilution (Trek Diagnostics, Cleveland, OH) according to the manufacturer’s instructions.

*Definitions*

Pneumococcal serotypes were classified as PCV10-types (serotypes 1, 4, 5, 6B, 7F, 9V, 14, 18C, 19F, 23F), PCV13-unique serotypes (serotypes contained in 13-valent pneumococcal conjugate vaccine [PCV13] but not in PCV10—3, 6A, and 19A), or non-vaccine types (NVT; all other serotypes). When more than one pneumococcal serotype was identified from a specimen, we classified participants as colonized with a vaccine serotype if ≥1 serotype was contained in the specific vaccine. We stratified the analyses by PCV10 dose (≥2 PCV10 doses versus ≤1 PCV10 dose) based on a systematic review that reported that ≥2 PCV doses in infants had impact on vaccine-type pneumococcal carriage [8]. Antibiotic susceptibility was determined using the 2012 Clinical and Laboratory Standards Institute criteria for minimum inhibitory concentrations (MIC) [6, 9, 10]. We used the oral breakpoints for penicillin and nonmeningitis breakpoints for ceftriaxone [6, 9]. Intermediate and resistant isolates were considered nonsusceptible to the antibiotic tested.

*Data management and analysis*

Sample sizes were calculated to detect a 45% reduction in vaccine-type carriage among children targeted for PCV10 vaccination (aged <1 year in Kibera and aged <5 years in Lwak) and adults living with HIV, and a 30% reduction in vaccine-type carriage in children who were not targeted for vaccination at the time the program began (aged 1–4 years in Kibera), assuming 80% power and alpha = 0.05. We performed descriptive analyses of participants by site, year, and age group (<1 year, 1–4 years, and adults). Chi-squared test or Fishers exact test were used to compare categorical variables and Wilcoxon Rank-Sum test was used for continuous variables. To calculate the 95% confidence interval (CI) for the annual pneumococcal carriage prevalence, we used the Wilson procedure for binomial distribution. We calculated unadjusted prevalence ratios using classic methods for estimation of risk ratios with 2009–2010 (2009 only in adults) as the reference period. Potential confounders associated with pneumococcal carriage as identified through previous studies were explored [4, 11-13]. Adjusted prevalence ratios were calculated using Poisson regression with robust error variance [14]. Changes in carriage prevalence by serotype were compared between 2009 and 2013 for children aged <5 years and for adults. We tested for changes in prevalence of the 13 vaccine- and 12 non-vaccine serotypes with highest frequencies post-PCV10. To account for multiple comparisons, we used a Bonferroni correction, where a *P* value of <.002 (.05/25) was considered statistically significant. Changes in the proportion (among pneumococcal isolates) and carriage prevalence (among survey participants) of antibiotic-nonsusceptible pneumococci between 2009 and 2013 were tested using chi-squared test or Fisher’s exact test. Analyses were performed using SAS software (version 9.4; SAS Institute, Cary, NC).

Supplementary Table 1. Characteristics of survey participants aged <5 years in Kibera by year, Kenya 2009–2013

|  | 2009  N = 539 | 2010  N = 256 | 2011  N = 402 | 2012  N = 414 | 2013  N = 462 |
| --- | --- | --- | --- | --- | --- |
| Females, n (%) | 276 (51.2) | 121 (47.3) | 197 (49.0) | 210 (50.7) | 249 (53.9) |
| Age <1 year, n (%) | 108 (20.0) | 99 (38.7) | 137 (34.1) | 128 (30.9) | 151 (32.7) |
| 0 PCV10 dose | — | — | 10 (7.5)^a^ | 4 (3.2)^b^ | 1 (0.7)^c^ |
| 1 PCV10 dose | — | — | 7 (5.2) ^a^ | 7 (5.7) ^b^ | 7 (4.8) ^c^ |
| 2 PCV10 dose | — | — | 21 (15.7) ^a^ | 14 (11.3) ^b^ | 16 (10.9) ^c^ |
| 3 PCV10 dose | — | — | 96 (71.6) ^a^ | 99 (79.8) ^b^ | 123 (83.7) ^c^ |
| Age 1–4 years, n (%) | 431 (80.0) | 157 (61.3) | 265 (65.9) | 286 (69.1) | 311 (67.3) |
| 0 PCV10 dose | — | — | 224 (86.5)^d^ | 168 (61.3)^e^ | 108 (37.9)^f^ |
| 1 PCV10 dose | — | — | 9 (3.5) ^d^ | 10 (3.7) ^e^ | 4 (1.4) ^f^ |
| 2 PCV10 dose | — | — | 7 (2.7) ^d^ | 8 (2.9) ^e^ | 7 (2.5) ^f^ |
| 3 PCV10 dose | — | — | 19 (7.3) ^d^ | 88 (32.1) ^e^ | 166 (58.3) ^f^ |
| Number of people sleeping in the same room as the child, median (IQR) | 5 (4–6) | 4.5 (3–6) | 5 (4–6) | 5 (4–6) | 5 (4–6) |
| Number of children in the household attending school or daycare, n (%) |  |  |  |  |  |
| 0 | 104 (19.4) | 51 (19.9) | 73 (18.2) | 66 (15.9) | 76 (16.5) |
| 1 or more | 432 (80.6) | 205 (80.1) | 329 (81.8) | 348 (84.1) | 386 (83.6) |
| Cough, fever, fast breathing, or pneumonia within 30 days, n (%) | 372 (69.0) | 184 (71.9) | 245 (61.0) | 315 (76.1) | 278 (60.2) |
| Tobacco smoke at home, n (%) | 59 (11.0) | 20 (7.8) | 38 (9.5) | 51 (12.3) | 44 (9.5) |
| Types of fuels used for cooking, n (%) |  |  |  |  |  |
| Fuelwood | 8 (1.5) | 8 (3.1) | 1 (0.3) | 2 (0.5) | 3 (0.7) |
| Charcoal | 508 (94.3) | 245 (95.7) | 389 (96.8) | 392 (94.7) | 434 (93.4) |
| Kerosene | 355 (65.9) | 218 (85.2) | 280 (69.7) | 257 (62.1) | 311 (67.3) |
| Others | 11 (2.0) | 15 (5.9) | 8 (2.0) | 12 (2.9) | 21 (4.6) |
| Area used for cooking, n (%) |  |  |  |  |  |
| Same area where you live or sleep | 486 (90.2) | 226 (88.3) | 387 (96.3) | 385 (93.0) | 439 (95.0) |
| All other sites | 53 (9.8) | 30 (11.7) | 15 (3.7) | 29 (7.0) | 23 (5.0) |
| Antibiotic use, n (%) |  |  |  |  |  |
| Current | 22 (4.1) | 13 (5.1) | 27 (6.7) | 22 (5.3) | 48 (10.4) |
| Use within 7 days of the survey | 87 (16.1) | 49 (19.1) | 66 (16.4) | 69 (16.7) | 108 (23.4) |
| Use within 30 days of the survey | 191 (35.4) | 117 (45.7) | 129 (32.1) | 148 (35.8) | 195 (42.2) |

Abbreviations: IQR, interquartile range

1. Out of 134 children with known vaccination history
2. Out of 124 children with known vaccination history
3. Out of 147 children with known vaccination history
4. Out of 259 children with known vaccination history
5. Out of 274 children with known vaccination history
6. Out of 285 children with known vaccination history

Supplementary Table 2. Characteristics of survey participants aged <5 years in Lwak by year, Kenya 2009–2013

|  | 2009  N = 188 | 2010  N = 173 | 2011  N = 148 | 2012  N = 177 | 2013  N = 203 |
| --- | --- | --- | --- | --- | --- |
| Females, n (%) | 101 (53.7) | 84 (48.6) | 68 (46.0) | 85 (48.0) | 100 (49.3) |
| Age <1 year, n (%) | 22 (11.7) | 18 (10.4) | 17 (11.5) | 25 (14.1) | 29 (14.3) |
| 0 PCV10 dose | — | — | 1 (5.9) | 1 (4.2)^a^ | 0 |
| 1 PCV10 dose | — | — | 3 (17.6) | 6 (25.0) ^a^ | 0 |
| 2 PCV10 dose | — | — | 2 (11.8) | 0 ^a^ | 4 (13.8) |
| 3 PCV10 dose | — | — | 11 (64.7) | 17 (70.8) ^a^ | 25 (86.2) |
| Age 1–4 years, n (%) | 166 (88.3) | 155 (89.6) | 131 (88.5) | 152 (85.9) | 174 (85.7) |
| 0 PCV10 dose | — | — | 21 (16.2) ^b^ | 19 (12.9) ^c^ | 8 (4.9) ^d^ |
| 1 PCV10 dose | — | — | 32 (24.6) ^b^ | 30 (20.4) ^c^ | 21 (12.8) ^d^ |
| 2 PCV10 dose | — | — | 68 (52.3) ^b^ | 59 (40.1) ^c^ | 35 (21.3) ^d^ |
| 3 PCV10 dose | — | — | 9 (6.9) ^b^ | 39 (26.5) ^c^ | 100 (61.0)^d^ |
| Number of people sleeping in the same room as the child, median (IQR) | 4 (3–4) | 3 (3–4) | 3 (2–4) | 3 (3–4) | 3 (2–4) |
| Number of children in the household attending school or daycare, n (%) |  |  |  |  |  |
| 0 | 33 (17.6) | 26 (15.0) | 20 (13.5) | 17 (9.6) | 22 (10.8) |
| 1 or more | 155 (82.5) | 147 (85.0) | 128 (86.5) | 160 (90.4) | 181 (89.2) |
| Cough, fever, fast breathing, or pneumonia within 30 days, n (%) | 115 (61.2) | 68 (39.3) | 112 (75.7) | 108 (61.0) | 101 (50.0) |
| Tobacco smoke at home, n (%) | 34 (18.2) | 29 (16.8) | 20 (13.6) | 32 (18.2) | 25 (12.3) |
| Types of fuels used for cooking, n (%) |  |  |  |  |  |
| Fuelwood | 180 (95.7) | 170 (98.3) | 142 (96.0) | 174 (98.3) | 196 (96.6) |
| Charcoal | 77 (41.0) | 66 (38.2) | 65 (43.9) | 86 (48.6) | 59 (29.1) |
| Kerosene | 7 (3.7) | 6 (3.5) | 4 (2.7) | 7 (4.0) | 8 (3.9) |
| Others | 1 (0.5) | 0 | 0 | 15 (8.5) | 6 (3.0) |
| Area used for cooking, n (%) |  |  |  |  |  |
| Same area where you live or sleep | 68 (36.2) | 47 (27.2) | 31 (21.0) | 53 (29.9) | 45 (22.2) |
| All other sites | 120 (63.8) | 126 (72.8) | 117 (79.1) | 124 (70.1) | 158 (77.8) |
| Antibiotic use, n (%) |  |  |  |  |  |
| Current use | 14 (7.5) | 15 (8.7) | 17 (11.5) | 17 (9.6) | 14 (6.9) |
| Use within 7 days of the survey | 45 (23.9) | 38 (22.0) | 33 (22.3) | 41 (23.2) | 42 (20.7) |
| Use within 30 days of the survey | 70 (37.2) | 62 (35.8) | 66 (44.6) | 71 (40.1) | 63 (31.0) |

Abbreviations: IQR, interquartile range

1. Out of 24 children with known vaccination history
2. Out of 130 children with known vaccination history
3. Out of 147 children with known vaccination history
4. Out of 164 children with known vaccination history

Supplementary Table 3. Characteristics of HIV-positive adult survey participants in Lwak by year, Kenya 2009, 2011–2013

|  | 2009  N = 549 | 2011  N = 423 | 2012  N = 526 | 2013  N = 530 | Total  N = 2,028 |
| --- | --- | --- | --- | --- | --- |
| Females, n (%) | 374 (68.1) | 266 (62.9) | 347 (66.0) | 362 (68.3) | 1,349 (66.5) |
| Median age (IQR) | 32 (27–37) | 34 (29–39) | 34 (29–39) | 34 (29–39) | 34 (29–39) |
| Aged <30 years, n (%) | 187 (34.1) | 114 (27.0) | 139 (26.4) | 141 (26.6) | 581 (28.7) |
| Aged 30–39 years, n (%) | 266 (48.5) | 212 (50.1) | 272 (51.7) | 263 (29.6) | 1,013 (50.0) |
| Aged ≥40 years, n (%) | 95 (17.3) | 97 (22.9) | 115 (21.9) | 126 (23.8) | 433 (21.4) |
| Employed, n (%) | 112 (20.5) | 52 (12.3) | 117 (22.3) | 61 (11.5) | 342 (16.9) |
| Number of children aged <5 years in the household, median (IQR) | 1 (1–2) | 1 (1–2) | 1 (1–2) | 1 (1–2) | 1 (1–2) |
| Cough, fever, fast breathing, or pneumonia within 30 days, n (%) | 273 (49.7) | 255 (60.3) | 241 (45.8) | 215 (40.6) | 984 (48.5) |
| Tobacco smoker, n (%) | 29 (5.3) | 23 (5.5) | 25 (4.8) | 15 (2.8) | 9 2 (4.6) |
| Tobacco smoke at home, n (%) | 99 (18.1) | 42 (10.0) | 53 (10.1) | 50 (9.4) | 244 (12.1) |
| Types of fuels used for cooking, n (%) |  |  |  |  |  |
| Fuelwood | 540 (98.4) | 412 (97.4) | 517 (98.3) | 515 (97.2) | 1,984 (97.8) |
| Charcoal | 207 (37.7) | 209 (49.4) | 264(50.2) | 193 (36.4) | 873 (43.1) |
| Kerosene | 20 (3.6) | 42 (9.9) | 18 (3.4) | 17 (3.2) | 97 (4.8) |
| Others | 4 (0.7) | 1 (0.2) | 30 (5.7) | 19 (3.6) | 54 (2.7) |
| Area used for cooking, n (%) |  |  |  |  |  |
| Same area where you live or sleep | 243 (44.3) | 81 (19.2) | 137 (26.1) | 127 (24.0) | 588 (29.0) |
| All other sites | 306 (55.7) | 342 (80.9) | 389 (74.0) | 403 (76.0) | 1,440 (71.0) |
| Antibiotic use, n (%) |  |  |  |  |  |
| Current use | 169 (30.8) | 200 (47.3) | 295 (56.1) | 302 (57.0) | 966 (47.6) |
| Use within 7 days of the survey | 313 (57.0) | 292 (69.0) | 394 (74.9) | 406 (76.6) | 1405 (69.3) |
| Use within 30 days of the survey | 357 (65.0) | 316 (74.7) | 419 (79.7) | 416 (78.4) | 1508(74.4) |
| Use of cotrimoxazole, n (%) |  |  |  |  |  |
| Current use | 161 (29.3) | 191 (45.2) | 287 (54.6) | 297 (56.0) | 966 (46.2) |
| Use within 7 days of the survey | 296 (53.9) | 274 (64.8) | 384 (73.0) | 395 (74.5) | 1,349 (66.5) |
| Use within 30 days of the survey | 298 (54.3) | 292 (69.0) | 404 (76.8) | 400 (75.5) | 1,394 (68.7) |

**Supplementary Figure. Vaccine implementation strategies during the first year and target age groups for cross-sectional surveys by site**


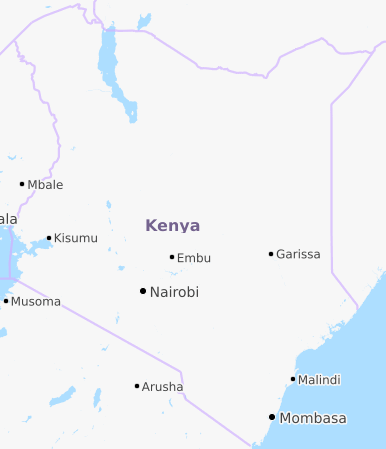

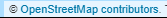


**Kibera**

**Lwak**

Lwak

- Vaccine implementation strategy during 2011
  - All children aged <1 year targeted to receive three doses
  - Catch-up vaccination targeting children aged 1–4 years (up to two doses of PCV10)
- Cross-sectional survey enrollment
  - Random sampling of children aged <5 years
  - Adults with children aged <5 years in household

Kibera

- Vaccine implementation strategy during 2011
  - All children aged <1 year targeted to receive three doses
  - No catch-up vaccination targeting children aged 1–4 years
- Cross-sectional survey enrollment
  - Age-stratified (<1 year and 1–4 years) random sampling
  - No adult enrollment

**References**

1. Feikin DR, Olack B, Bigogo GM, et al. The burden of common infectious disease syndromes at the clinic and household level from population-based surveillance in rural and urban Kenya. PLOS one **2011**; 6(1): e16085.

2. Feikin DR, Audi A, Olack B, et al. Evaluation of the optimal recall period for disease symptoms in home-based morbidity surveillance in rural and urban Kenya. Int J Epidemiol **2010**; 39(2): 450-8.

3. Dalal W, Feikin DR, Amolloh M, et al. Home-based HIV testing and counseling in rural and urban Kenyan communities. Journal of acquired immune deficiency syndromes (1999) **2013**; 62(2): e47-54.

4. Conklin LM, Bigogo G, Jagero G, et al. High Streptococcus pneumoniae colonization prevalence among HIV-infected Kenyan parents in the year before pneumococcal conjugate vaccine introduction. BMC infectious diseases **2016**; 16(1): 18.

5. Adazu K, Lindblade KA, Rosen DH, et al. Health and demographic surveillance in rural western Kenya: a platform for evaluating interventions to reduce morbidity and mortality from infectious diseases. The American journal of tropical medicine and hygiene **2005**; 73(6): 1151-8.

6. Kobayashi M, Conklin LM, Bigogo G, et al. Pneumococcal carriage and antibiotic susceptibility patterns from two cross-sectional colonization surveys among children aged <5 years prior to the introduction of 10-valent pneumococcal conjugate vaccine - Kenya, 2009-2010. BMC infectious diseases **2017**; 17(1): 25.

7. Carvalho MDG, Pimenta FC, Jackson D, et al. Revisiting pneumococcal carriage by use of broth enrichment and PCR techniques for enhanced detection of carriage and serotypes. Journal of clinical microbiology **2010**; 48(5): 1611-8.

8. Fleming-Dutra KE, Conklin L, Loo JD, et al. Systematic review of the effect of pneumococcal conjugate vaccine dosing schedules on vaccine-type nasopharyngeal carriage. The Pediatric infectious disease journal **2014**; 33 Suppl 2: S152-60.

9. Clinical and Laboratory Standards Institute. Performance Standards for Antimicrobial Susceptibility Testing; Twenty-Second Informational Supplement. CLSI Document M100-S22. Wayne, PA: Clinical and Laboratory Standards Institute, **2012**.

10. Conklin LM, Bigogo G, Jagero G, et al. High Streptococcus pneumoniae colonization prevalence among HIV-infected Kenyan parents in the year before pneumococcal conjugate vaccine introduction. BMC infectious diseases **2016**; 16: 18.

11. Abdullahi O, Nyiro J, Lewa P, Slack M, Scott JA. The descriptive epidemiology of Streptococcus pneumoniae and Haemophilus influenzae nasopharyngeal carriage in children and adults in Kilifi district, Kenya. The Pediatric infectious disease journal **2008**; 27(1): 59-64.

12. Abdullahi O, Karani A, Tigoi CC, et al. The prevalence and risk factors for pneumococcal colonization of the nasopharynx among children in Kilifi District, Kenya. PloS one **2012**; 7(2): e30787.

13. Verani JR, Massora S, Acacio S, et al. Nasopharyngeal carriage of Streptococcus pneumoniae among HIV-infected and -uninfected children <5 years of age before introduction of pneumococcal conjugate vaccine in Mozambique. PloS one **2018**; 13(2): e0191113.

14. Zou G. A modified poisson regression approach to prospective studies with binary data. American journal of epidemiology **2004**; 159(7): 702-6.
